# Advancing the capability approach to well-being pathways from access and use of urban green space

**DOI:** 10.1038/s41598-026-63359-5

**Published:** 2026-07-20

**Authors:** Irvanu Rahman, Somidh Saha, Armin Grunwald

**Affiliations:** 1 Institute for Technology Assessment and Systems Analysis, Karlsruhe Institute of Technology, Karlstraße 11, 76133 Karlsruhe, Germany; 2 Industrial Engineering Department, Faculty of Engineering, Universitas Indonesia, Kampus UI Depok, 16424 Depok, Indonesia; 3 Institute for Geography and Geoecology, Karlsruhe Institute of Technology, Karlsruhe, Germany

**Keywords:** Environmental social sciences, Environmental studies, Geography, Geography

## Abstract

Urban green spaces (UGS) are critical for well-being, yet greening efforts often prioritize physical proximity while assuming equal benefit. Challenging this assumption, we apply Amartya Sen’s Capability Approach by conceptualizing and empirically assessing residents’ access-use-benefit pathways that determine their ability to convert green space availability into realized well-being. Using participatory survey data from Jakarta, we evaluated how travel time, transport modes, and socioeconomic conditions shape individuals’ ability to convert spatial access into realized engagement. We operationalize this pathway through a novel time-based benefit–cost ratio approach that measures constrained functioning and reveals inequalities hidden by proximity-based planning. Our results showed that residents with lower socioeconomic status and those reliant on walking or public transport face greater time burdens and constrained access, limiting their ability to benefit from the park meaningfully. These findings demonstrate that spatial proximity alone is insufficient for equitable greening outcomes. To support just and inclusive cities, planners must address structural and temporal barriers hindering the meaningful green space use and the well-being opportunities it enables, particularly those stemming from socioeconomic disadvantage, time constraints, and mobility limitations.

**Supplementary Information:**

The online version contains supplementary material available at 10.1038/s41598-026-63359-5.

## Introduction

Urban green spaces (UGS)—defined as all urban land covered by vegetation, including parks, gardens, greenways, and other vegetated areas in both public and private settings^1^—are increasingly recognized as critical infrastructure for building sustainable, healthy, and climate-resilient cities^2^. They contribute to physical and mental well-being, foster social interaction, and provide ecological services, including heat mitigation and air purification^3,4^. As a result, accessible green spaces have become central to urban sustainability agendas worldwide, particularly within the Sustainable Development Goals (SDG 11.7) and climate adaptation frameworks^2,5^.

Despite their importance, efforts to promote equitable green space access often rely on provision- or proximity-based metrics^6^, measured in per capita park area or distance thresholds^4^. While helpful in planning^7^, these approaches assume that the physical availability of resources automatically translates into use and that spatial distribution is an adequate proxy for equity^8^. In response, environmental justice (EJ) literature has advanced an alternative framework that moves beyond distributional metrics by emphasizing recognition of marginalized groups^9,10^.

Recognition justice focuses on whether diverse identities, needs, and experiences are visible and valued in environmental planning and governance^9,10^. It expands the concept of equity by emphasizing the social and cultural dimension of exclusion in green space access, drawing attention to whose values are prioritized in public space design and whose experiences are often excluded^11^. While this perspective expands the scope of justice beyond resource distribution and helps highlight who is marginalized, it leaves open important questions about how and why certain groups continue to be underserved^9,11^. This limitation points to the next EJ dimension, participation, which concerns who has a voice in environmental decision-making and whether planning processes are inclusive, transparent, and responsive^9^.

Participation shifts the justice perspective to governance processes, raising concerns about who is included in decision-making and whether planning structures genuinely reflect diverse voices and lived realities^9^. In UGS contexts, it was typically associated with community engagement in park design, planning consultations, or co-governance models^11^. While such mechanisms were vital for procedural fairness, they often proved ineffective when embedded within broader governance systems that systematically privileged economic interests and advantaged social groups^12,13^. This concern aligns with Nancy Fraser’s^10^ criticism of justice misframing, where participation was confined to procedural forms without addressing the redistribution of power and influence required to produce equitable outcomes. This tension is particularly relevant in rapidly urbanizing cities in the Global South, such as Jakarta, Indonesia.

In Jakarta, participatory mechanisms in green space planning were often framed as stewardship—emphasizing caretaking and responsibility—rather than governance^14^, thereby positioning citizens in symbolic roles rather than active decision-makers. Public involvement tended to be limited to peripheral consultation, with minimal influence over critical aspects such as park location, amenity design, or long-term management^14^. These gestures unfolded within a broader development agenda shaped by elite planning interests and land market dynamics, which prioritized economic value over social inclusiveness^14,15^. As a result, parks with rich cultural and recreational amenities were often concentrated in wealthier neighborhoods, while residents in lower-income neighborhoods faced persistent spatial and infrastructural disadvantages^16^.

These patterns raise two critical limitations. First, participation functions less as a vehicle for transformative empowerment and more as a means of legitimizing preconfigured agendas, revealing the limits of procedural inclusion in addressing the structural injustices embedded in Jakarta’s urban governance. Second, as access to green spaces was mediated by socioeconomic status^16^, the practical effort required to reach them varied significantly across population groups, even when they were technically accessible. While location determines where green spaces are situated, it remains an important open question whether they are equitably usable by all urban residents.

Previous studies in the EJ domain treated proximity to parks as a proxy for equitable access^6,7^. Yet, growing evidence suggested that health and well-being benefits were primarily generated by actual patterns of use and engagement rather than by mere spatial availability^17^. While closer residential proximity to parks was generally associated with better health outcomes, these relationships were consistently contingent on whether individuals perceive the space as safe, high-quality, and socially welcoming, as well as on their personal mobility and life circumstances^18^. These studies suggest that proximity to parks or their availability does not guarantee whether people can meaningfully use the spaces and obtain health benefits from them. The disconnect between nominal access and realized use underscores a critical gap in the prevailing EJ literature and existing planning approaches, raising broader questions about how varying access efforts translate into meaningful opportunities to use and benefit from green spaces. This highlights the need for an analytical framework that evaluates UGS equity through the lived experiences of diverse communities.

Responding to these limitations, this study operationalizes Amartya Sen’s Capability Approach to reframe environmental justice in terms of individuals’ freedoms^19^, specifically their capacity to convert access into the meaningful use of green spaces. We define access and meaningful use as the ability to transform green space availability into actual engagement, determined by whether the effort invested in reaching a preferred park is justified by the time spent there. This framing positions travel effort and agency as conversion factors shaped by socioeconomic status and mobility conditions, conceptualizing green space not merely as physical amenities but as a site of potential functioning. We therefore shift the justice lens from *who has access* to *who can use and benefit from the spaces that matter to them.* This capability-based perspective is particularly relevant in Jakarta’s fragmented landscape, where structural positioning and infrastructural inequalities significantly shaped residents’ ability to benefit from green spaces^16^. We follow Sen’s open and context-sensitive formulation, emphasizing conversion factors and individual diversity as central dimensions in diagnosing potential inequality in green space use.

**Fig. 1:**
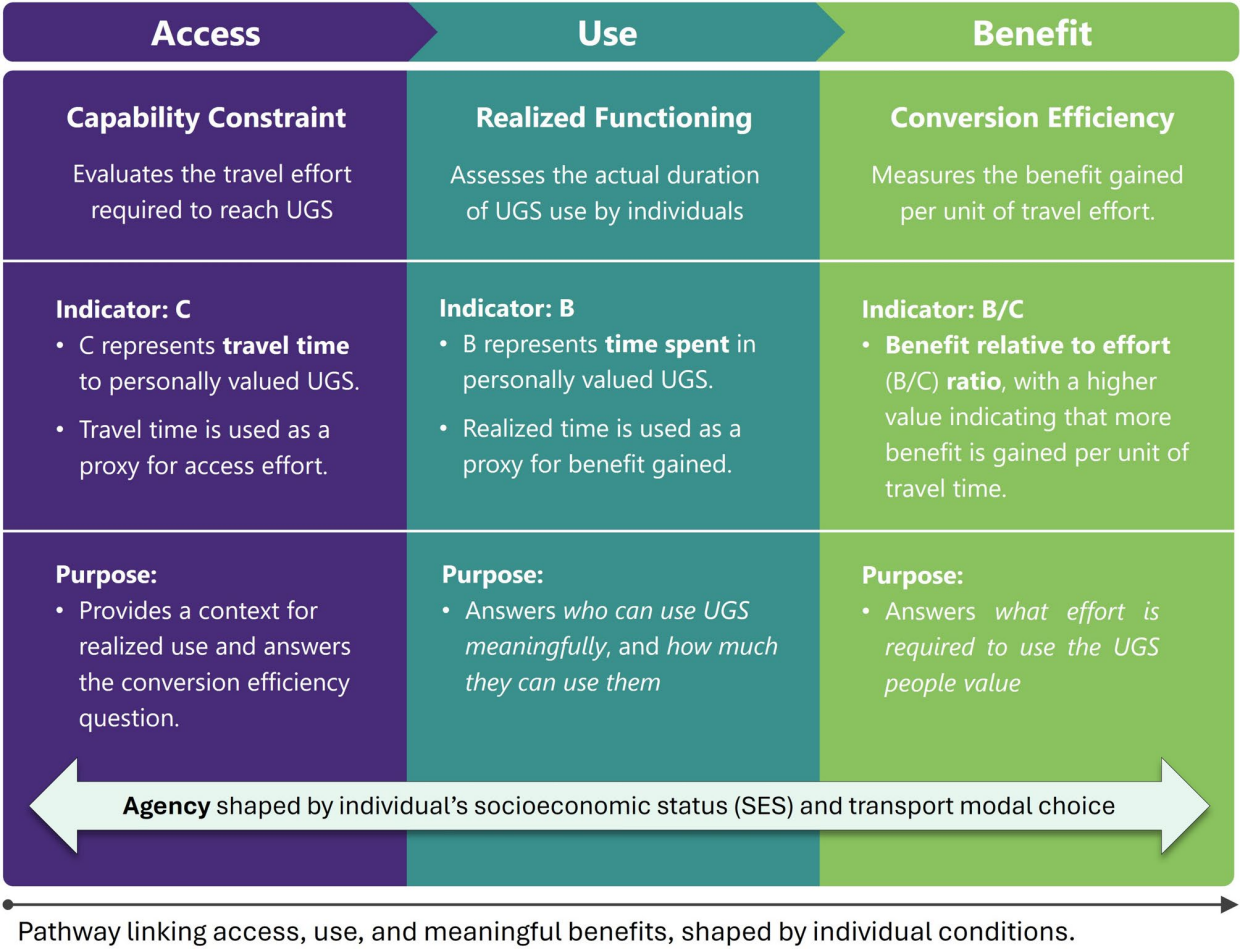
A capability-based framework for the individual well-being pathway from urban green space (UGS). The framework links access, use, and benefit through three analytical sequences: capability constraints, realized functioning, and conversion efficiency. These dimensions are shaped by individual socioeconomic status (SES) and transport mode, revealing how barriers to time, mobility options, and opportunity determine meaningful engagement with green space.

Building on this conceptual foundation, this study operationalizes the Capability Approach as a normative justice framework through a temporal benefit-cost (B/C) framework (Fig. 1) that investigates individuals’ capability to convert available green space opportunities into realized use and health-related benefits. This ratio compares the benefit—time spent in a self-identified, personally meaningful park—to the cost, measured as the travel time required to reach it. Drawing on empirical data from our previous study^16^, we analyze time-related constraints and conversion factors for 377 residents who identified their preferred parks, reported visit frequency and duration, travel mode, and socioeconomic status of their residence. Since each respondent chose a park they consider valuable, we treat each location as equally meaningful to the individual who selected it and do not compare one park with another. This enables assessment of whether individuals from different socioeconomic contexts have practical opportunities to benefit from the green space they value.

## Results

### Capability constraints in mobility and access by socioeconomic status

**Fig. 2:**
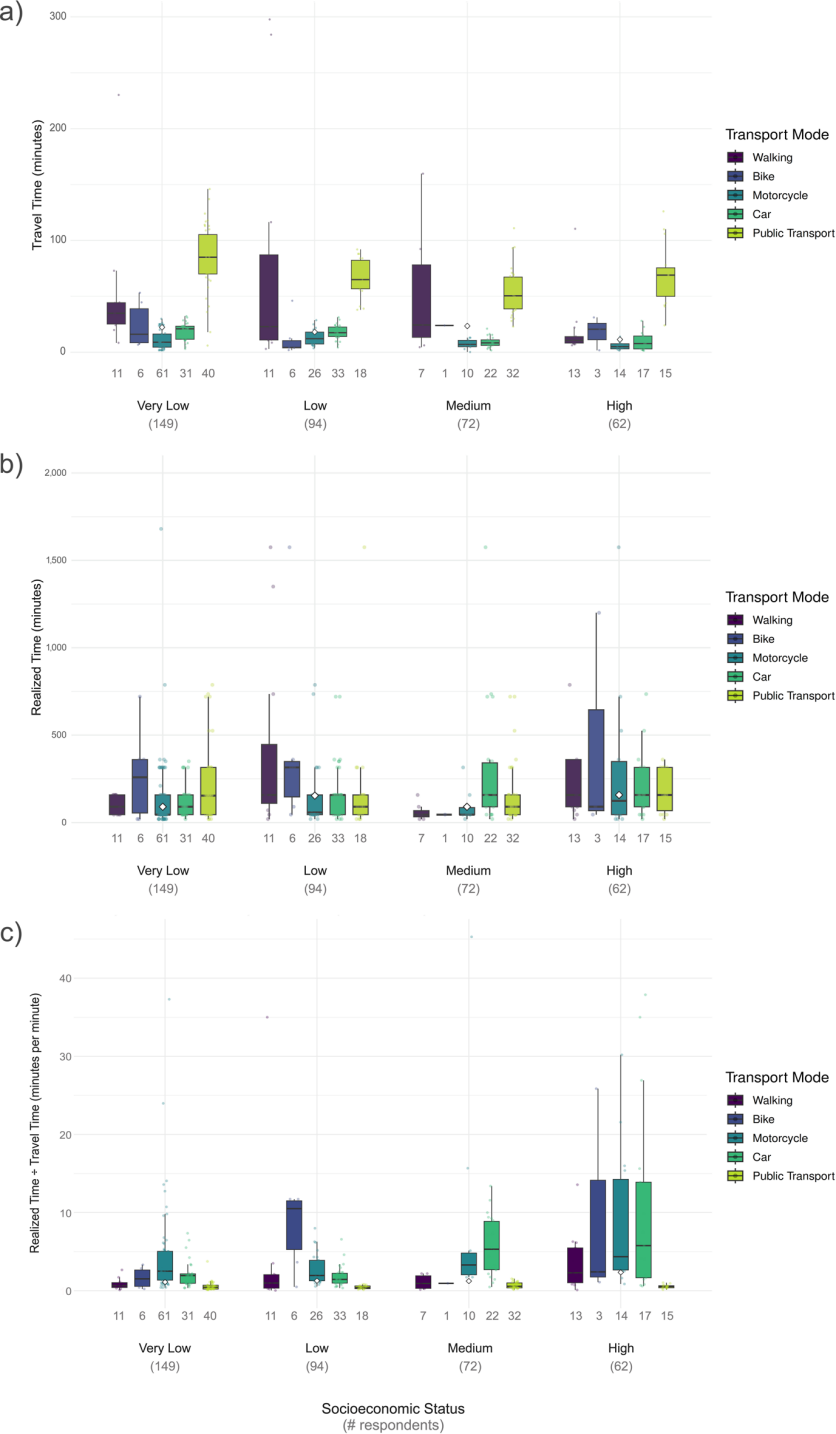
Boxplots summarizing three dimensions of green space access and use across SES and transport modes (n = 377). (**a**), travel time for a round trip to the preferred park across the SES groups. (**b**), realized time captures the actual duration of time spent in the preferred park, indicating how access translates into functioning. The Y-axis is limited to 2,000 min for visual clarity; the full distribution is shown in Supplementary Fig. 1. (**c**), conversion efficiency (benefit-per-cost ratio), highlights disparities in how travel effort leads to meaningful use. Each box represents the time distribution within SES groups (Very Low, Low, Medium, High) for each mode of transport. Numbers below each box indicate the number of respondents using that transport mode; numbers in parentheses indicate the total number of respondents in each SES group. The central diamond marks the median, boxes represent the interquartile range (IQR, 25^th^–75^th^ percentile), and whiskers extend to 1.5 × IQR. Outliers beyond the whiskers are plotted as individual points. Jittered dots represent individual observations across SES and mode.

Significant variation in travel times was observed across transport modes and socioeconomic groups (Fig. 2a). Respondents using motorized transport, such as cars and motorcycles, consistently reported shorter and more predictable travel times across all socioeconomic statuses (SES). In contrast, respondents who walked or used public transport reported longer travel times, with wider dispersion across individuals, particularly among those from Low, Medium, and Very Low SES groups. A notable travel time exceeding 200 min was observed among a few individuals in the Low SES group.

### Realized functioning of green space use: usage, perceived benefits, and time realization

**Fig. 3:**
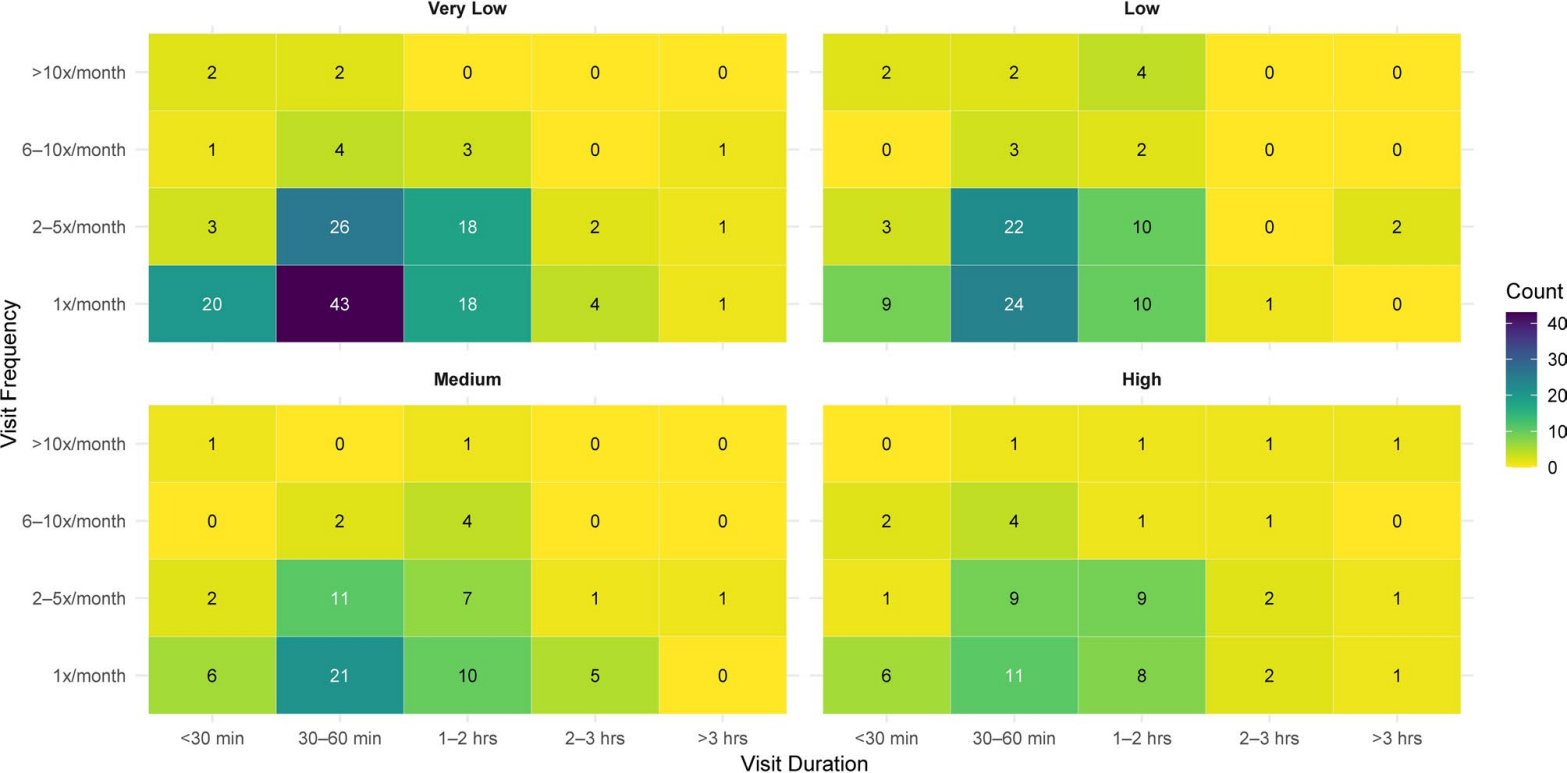
Green space usage heatmap matrix showing the distribution of respondents (n = 377) by visit frequency and visit duration across SES groups. Each cell represents the number of respondents falling within a specific frequency-duration combination. A darker color reflects a higher number of respondents.

To explore realized functioning, we examined how green space use varies across socioeconomic groups by plotting visit frequency against duration in a heatmap (Fig. 3). From the heatmap, park visits were concentrated in moderate patterns across all groups, particularly within the 30–60-min duration, occurring 1 to 5 times per month. Very Low SES respondents were most heavily clustered in the lowest usage category (one visit per month, 30–60 min), with minimal representation in higher-frequency or longer-duration cells. The intensive use of green space was uncommon across groups, suggesting a limited ability to extend that engagement to more frequent visits.

**Fig. 4:**
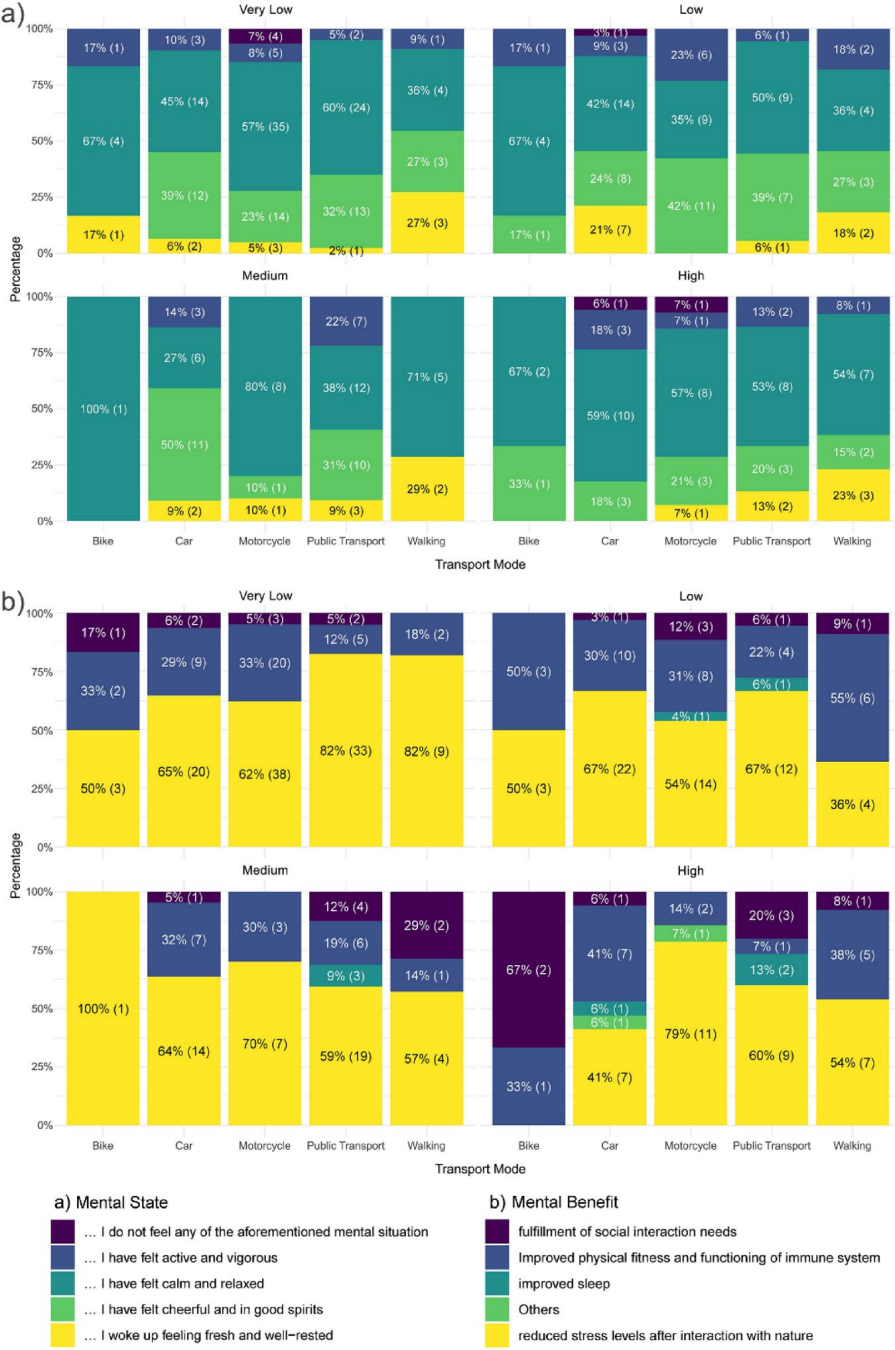
Reported mental state and benefits of green space use (n = 377). Distribution of a, self-reported mental states, and b, perceived mental benefits disaggregated by SES group and transport mode.

We then illustrated how these usage patterns translated into perceived mental states and benefits (Fig. 4). Across SES and transportation groups, respondents consistently reported benefits to their cultural and mental health, including reduced stress, relaxation, and improved mood. Differences were minor, indicating that the perceived benefits are broadly similar once individuals reach parks. Given this similarity, the subsequent analysis examined monthly realized time, calculated as the product of the reported monthly visit frequency and the visit duration, to capture variation of time spent in green space across SES groups.

The boxplot of realized time (Fig. 2b) revealed notable differences across SES groups and transport modes. High SES respondents showed the widest variation, particularly among bikers and public transport users, with several outliers exceeding 2,000 min per month, reflecting a higher ceiling of realized functioning. In contrast, Low SES respondents exhibited narrower, more compressed distributions, suggesting constrained use. Very Low SES individuals showed slightly more variation, especially among bikers and public transport users, with a few achieving extended durations. Both Low and Very Low SES groups displayed tighter, lower distributions for motorcycle and car users. Medium-SES respondents showed relatively compressed realized time across modes, particularly among walking, biking, and motorcycle users. These patterns appeared more uniform, partly due to the smaller number of respondents in this group, which limits the variability captured. However, public transport users displayed slightly greater heterogeneity in realized time than individuals in other SES groups.

### Barriers to converting access to green space use

To examine how well individuals across SES groups convert travel effort into green space use, we constructed a box plot of the benefit-to-cost (B/C) ratio, where B represented realized time spent in the valued park (benefit), and C represented travel time as the effort required to reach it (cost). This measure provided a nuanced view of access inequality in terms of opportunity and how that opportunity was translated into actual well-being outcomes, revealing who gained the most and the least from their travel efforts. Higher B/C values reflected more efficient conversion or greater benefit per unit of effort, while lower values indicated constrained agency, where physical access did not lead to meaningful use.

The benefit-per-effort (B/C) ratio (Fig. 2c) revealed stark disparities in conversion efficiency by SES and transport mode. High SES respondents, particularly those who used cars and motorcycles, consistently exhibited higher and more flexible conversion efficiencies. In contrast, Very Low and Low SES groups generally displayed lower median B/C values, indicating substantial constraints. Notably, biking yielded elevated B/C ratios among a minority of Low SES respondents, while motorcycle and car users occasionally achieved high-efficiency outcomes, suggesting individual adaptability in overcoming access barriers. Medium-SES respondents exhibited fragmented patterns, with low median values resembling those of lower-SES groups, but greater variability among car and motorcycle users, reflecting modest conversion capabilities under specific conditions. Across all SES groups, public transport consistently had the lowest conversion efficiency, severely limiting residents’ ability to translate access to green space into meaningful use.

**Fig. 5:**
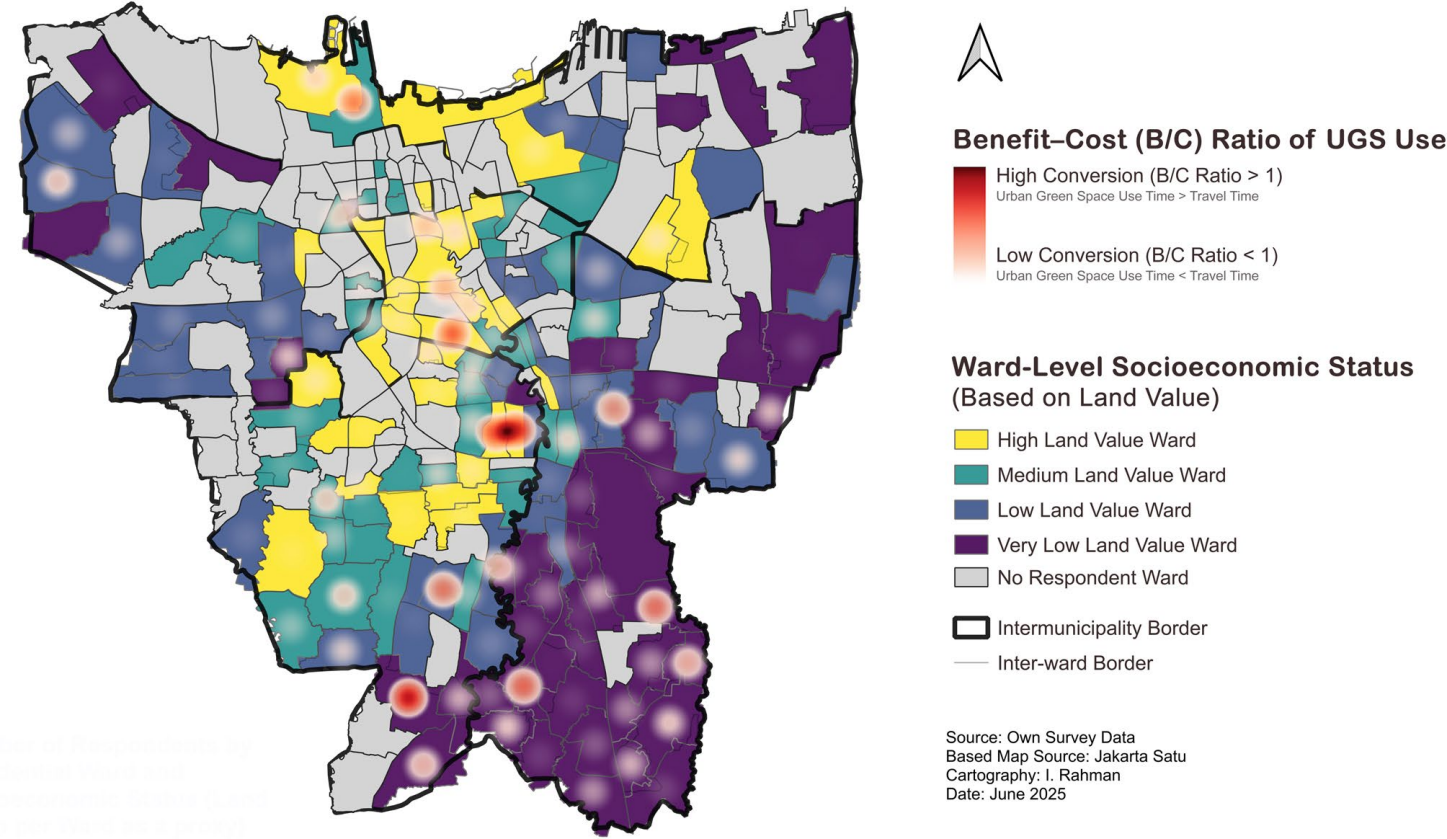
Conversion efficiency heatmap across ward-level socioeconomic distributions. The Benefit–Cost (B/C) ratio of green space use is overlaid on ward-level socioeconomic status, classified by land value. Red hotspots indicate high conversion efficiency (more time spent in green space relative to travel time), while pale zones indicate low benefit relative to the effort of accessing green space. Wards shown in gray were not represented in the survey sample.

A heatmap of the B/C ratio was overlaid on ward-level SES classifications to reveal spatial disparities in conversion efficiency (Fig. 5). The highest-efficiency zones cluster in two affluent wards of South Jakarta, where residents make more effective use of parks relative to their travel effort. Several medium- and low-value wards in the Southern part of the city also showed pockets of efficiency, while many wealthy wards in central and northern areas displayed lighter hotspots, suggesting poor conversion despite affluence.

## Discussion

This study reframes UGS equity by applying Sen’s Capability Approach^19^ to advance an individual-centered justice framework focused on real freedoms to access, use, and benefit from urban nature. Moving beyond spatial proximity or availability-based assessments of access, we introduce a conversion-based lens to diagnose how structural conditions shape the ability to translate access into meaningful use. By examining how individuals reach, engage with, and derive well-being from green spaces they value, our analysis reveals patterns of inequality shaped by their socioeconomic status and transportation options, through three analytical stages of the Capability Approach.

First, travel time patterns revealed uneven mobility burdens in reaching preferred parks across SES groups. Low- and very low-SES respondents faced the greatest barriers, relying on walking, biking, or public transportation, thereby prolonging their journeys and reflecting constrained mobility choices. In contrast, higher-SES individuals benefited from shorter and more consistent travel times through motorized options. Yet across all SES groups, public transport users encountered persistent time barriers, exposing structural limitations in Jakarta’s mobility system. These findings align with broader mobility justice debates, showing how transport infrastructures systematically privilege some groups over others^20^. As Michelangeli et al.^21^ demonstrated that spatial provision alone cannot deliver equal outcomes without equitable connectivity, while van Burgsteden et al.^22^ highlighted the role of reliable transportation in pursuing well-being. Taken together, our results suggest that the exclusions faced by lower-SES and non-motorized users are not mere inefficiencies but systemic asymmetries that constrain agency, highlighting transportation’s role as capability-enabling infrastructure—a structural prerequisite for achieving well-being outcomes from green spaces.

Second, realized functioning patterns reveal that structural constraints persist beyond access. While individuals expressed preferences for park engagement, such as timing, duration, frequency, and mode of transport, the freedom to realize those preferences was unequally distributed. In Jakarta, high-SES individuals not only had access in theory but also the practical conditions to use green spaces for longer and in more varied ways. By contrast, low-SES individuals exhibited fragile functioning with narrower, more predictable patterns, suggesting structurally limited use. Medium-SES groups fell in between: their engagement was regular but confined to shorter, more uniform durations, indicating constrained functioning without expanded freedom. Taken together, these disparities highlight that the key barrier is not simply spatial distance but the scarcity of discretionary time and leisure opportunities, reinforcing Goodin et al.’s^23^ argument that time poverty can be more limiting than physical access.

The disparities in realized functioning lead to the final equity inquiry in our Capability Approach analysis: conversion efficiency. The benefit-per-cost (B/C) captures how efficiently individuals convert travel effort into time spent at their preferred park. High-SES individuals generally achieved the highest and most stable ratios, reflecting their flexibility in maximizing benefits upon arrival, while low- and very low-SES respondents showed lower and more variable outcomes, with occasional high-efficiency outliers from bike or motorcycle use. These exceptions were relevant to what Lucas^24^ conceptualized as temporary coping mechanisms for residents with transport disadvantage. While they may ease immediate constraints, their lack of structural support renders them fragile and leaves their functionings vulnerable to disruption. Medium-SES groups occupy an unstable middle ground: their outcomes clustered in the mid-zone, suggesting functioning was possible but remained precarious. They may have physical access to the parks they value, yet their ability to convert that access into meaningful, repeatable engagement remains constrained. Such circumstances risk rendering them structurally invisible in planning, as they are too affluent to be prioritized and too constrained to fully benefit, revealing a key justice gap.

Across all SES groups, transport mode emerges as the decisive factor in shaping conversion efficiency. Public transport users consistently registered low B/C outcomes due to long travel times, poor last-mile connections, and limited flexibility—confirming Martens’s^25^ argument that transport justice must be evaluated by actual accessibility rather than provision alone. Conversely, private or on-demand motorized transport dramatically raised conversion efficiency, even for lower-SES groups. While these options serve as a powerful equalizer, they also highlight individual coping strategies for navigating fragmented systems and the absence of inclusive structural solutions^24^, underscoring the need for more coordinated accessibility planning and systemic design that aligns mobility services with land-use policies^26^.

A heatmap of B/C ratios further contextualizes these inequalities by showing that high efficiency emerges only under favorable alignments, as in two affluent wards of South Jakarta. Yet some equally wealthy wards underperform, while a few disadvantaged wards succeed unexpectedly. These patterns illustrate what we term a *conditional conversion advantage*: the effective realization of capability depends on the convergence of agency, spatial opportunity, and temporal flexibility. This observation reinforces Schwanen and Wang’s^27^ argument that well-being is shaped not only by where people are located but also by when and under what circumstances they can act. Given that temporal and contextual factors are as critical as spatial ones, this conditionality also aligns with insights from van Wee and Geurs^26^ and Lucas^24^ who each emphasize in different ways that accessibility inequalities arise not merely from spatial provision, but from the misalignment of infrastructure, time constraints, and individuals’ capacity to convert access into real opportunities.

By reframing access as a function of lived experience, this study challenges the limits of prevailing justice triptychs. While spatial provision, participation, and recognition remain essential, they are insufficient for achieving equity. The Capability Approach adds conceptual depth by grounding equity in lived realities, shifting the focus to the conversion gap: the disconnect between nominal access and actual use. The B/C metric, combined with SES-transport disaggregation, offers an operational framework for diagnosing this gap. Unlike top-down models that assess equity through modeled potential access (e.g., Nahmias-Biran & Shiftan^28^), our approach draws on bottom-up realities by using self-reported data to trace effort, usage, and benefits. By holding park desirability constant, the method isolates land value as the structural factor that conditions access, thereby shaping differential conversion outcomes.

In revealing variation in functioning under unequal conditions, the B/C metric exposes structural inequalities embedded in everyday mobility and access patterns that aggregate indicators often overlook. This contribution addresses a conceptual divide in the capability-based accessibility literature: the distinction between top-down assessments of transport and land-use systems and bottom-up evaluations of individual functioning, as identified by Vecchio and Martens^29^. One key caveat is that while these ratios provide an observed indicator of conversion efficiency, they are not a direct measure of an individual’s intrinsic capability, as some individuals may choose not to engage with available green space opportunities.

Moreover, our analysis contributes conceptually through the notion of a *conditional conversion advantage*. Empirically demonstrated by the B/C heatmap, this concept explains why affluence or proximity to parks does not automatically guarantee equitable conversion outcomes, which only materialize when such advantages are combined with enabling temporal and mobility conditions. From a policy perspective, the conversion efficiency heatmap translates subjective barriers into actionable, spatially explicit insights, allowing planners to identify clusters of suppressed freedom. It enables place-based diagnosis and informs spatial targeting of interventions, where mobility upgrades, park programming, or temporal flexibility are most needed. By bridging the gap between individual-level constraints and policy design, this helps address a common critique of the Capability Approach regarding its operationalization and scalability in public policymaking^30,31^.

Two key policy insights emerge. First, expanding green space alone will not achieve equity without addressing transport and temporal accessibility. This risk is particularly salient in Jakarta, where access to high-value parks is already socioeconomically stratified^16^. Our findings show that inequality is compounded: the injustice lies not only in whether parks are reachable, but also in whether they can be used meaningfully and under fair conditions. Motorized transport can enhance conversion efficiency, but reliance on it is neither equitable nor a viable long-term solution, given its broader health, safety, and environmental impacts. More inclusive public transport, with stronger first- and last-mile connections with integrated modes, is key to equitable access and meaningful green space use.

Second, medium-SES individuals require greater attention in equity planning. Often overlooked, they face rigid constraints in accessing and benefiting from green spaces, yet are neither as affluent nor as prioritized as low-SES groups. This hidden exclusion is particularly salient in Jakarta’s sustainability strategies, which prioritize infrastructure expansion but risk reproducing inequities if structural constraints are overlooked. Addressing these barriers requires time-sensitive measures, such as extended city-wide park hours, workplace incentives, and micro-parks along residential or employment corridors, to reduce opportunity costs and support incidental use, particularly for those with inflexible routines. Such interventions not only advance local equity goals but also contribute directly to SDG 11.7’s mandate to ensure universal access to safe, inclusive, and accessible green spaces. Yet our findings suggest that achieving this global agenda requires moving beyond provision to account for temporal and mobility constraints in planning, so that the freedom to use parks is secured alongside their spatial availability.

### Conclusion and limitations

This study presents one of the earliest empirical efforts to operationalize the Capability Approach for diagnosing urban green equity, introducing a transferable time-based benefit–cost (B/C) ratio that captures constrained functioning and offers a replicable tool for justice-oriented planning. By linking the realized use of green space to the effort required to reach it, the approach moves beyond availability-based access measures to demonstrate how mobility, time, and socioeconomic conditions jointly determine who can meaningfully benefit from urban nature. Demonstrated through the case of Jakarta yet generalizable across urban contexts, the framework advances urban sustainability research by reframing equity not as the presence of green space, but as the substantive freedom to use and derive well-being from it.

While grounded in a robust spatial-access dataset, the analysis is limited by proxy indicators, the absence of longitudinal coverage, and the lack of personal motivations, type of recreational preferences, and qualitative data on autonomy, safety, and comfort. Moreover, although the ward-level B/C heatmap addresses concerns about the Capability Approach’s policy applicability by translating individual constraints into actionable insights, it does not substitute for comprehensive capability measurement frameworks. Individuals may differ in their recreational preferences or access to alternative leisure opportunities, which could influence whether they consider visiting available parks. Instead, the contribution of this study lies in offering a diagnostic lens that foregrounds conversion factors, such as mobility systems, time poverty, and socioeconomic status (SES), as key influences shaping equity outcomes in practice.

From an accessibility measurement perspective, we recognize that using postcode centroids to represent residential locations introduces positional uncertainty in individual-level travel time estimation, given variations in postcode area sizes and respondents’ actual residential locations within those areas. This limitation is particularly relevant for public transport, where access depends on proximity to specific network entry points (e.g., bus stops, stations) and can vary significantly within a single postcode unit. However, this approach was chosen as a pragmatic compromise in ensuring comparability of origin–destination travel time estimates across respondents, as precise residential addresses could not be collected through the participatory survey due to ethical and data protection considerations.

Even as a temporal snapshot, the findings show how time-based diagnostics can uncover structural disparities in lived access and reframe sustainability beyond spatial availability toward enabling real opportunities for well-being. Future research should extend this approach by examining how governance arrangements and institutional dynamics sustain UGS inequality, even when well-intentioned greening interventions are in place, and by incorporating richer dimensions of capability, including personal leisure preferences, autonomy, perceived safety, and cultural belonging. By integrating these perspectives, capability-informed equity frameworks can strengthen their role as practical tools for advancing urban and environmental justice in rapidly urbanizing contexts such as Jakarta.

## Methods

### Survey framework and dataset origin

This study used a secondary analysis of an anonymous participatory mapping survey that examined individuals’ access to and use of green space in Jakarta, collected for a previous study^16^. The survey was designed to exclude any identifiable or sensitive personal information and to comply with the data protection guidelines of the Karlsruhe Institute of Technology (KIT). The survey design and data handling procedures were reviewed by KIT’s Data Protection Officer and recorded in the university’s electronic processing register (eVV) to ensure compliance with the requirements of the General Data Protection Regulation (GDPR). Participation in the survey was voluntary, and informed consent was obtained through the consent statement presented at the beginning of the questionnaire. Responses from individuals under 18 were also excluded from the dataset to ensure legal compliance. According to the Ethical Principles of KIT and the associated eVV risk assessment, the study was classified as minimal risk; therefore, formal approval from the KIT Ethics Committee was not required. All methods were carried out in accordance with relevant guidelines and regulations.

During the data collection, respondents were asked to identify one personally valued park, which serves as the destination for travel analysis. Each entry also included a residential postal code, which was used to determine the respondent’s ward of residence. In Jakarta, each postcode corresponds to a single ward, with a median area of 2.48 km^2^ (range: 0.27–15.63 km^2^) and a median population of 42,133 residents^32^. The centroid of that ward was then used as a proxy for the origin point in calculating travel effort. In total, 386 responses were retained, each providing sufficient data on destination, origin, and visitation patterns (visit frequency and duration per visit), forming the empirical foundation for analyzing realized access and use. While these indicators captured key dimensions of capability constraint and realized functioning discussed here, they may also be shaped by broader contextual factors (e.g., income, occupation, household responsibilities) that were not the focus of this study. Supplementary Table 1 provides the linkages between our survey-based and derived variables used in this study’s analysis and the corresponding Capability Approach concepts.

### Socioeconomic and spatial contexts of origins and valued park destinations

Supplementary Fig. 2 contextualizes the empirical basis of this study. Panel (**a**) showed the distribution of 386 respondents across Jakarta’s wards, with the dot size representing the number of respondents per ward and the color indicating ward-level land value classifications. Land value information, obtained from the Jakarta Provincial City Government^33^, was used here as a proxy for socioeconomic status^34^. It was categorized using a percentile-based threshold following the rationale established in our previous study^16^. This use of the land value conceptually aligns with Vecchio and Martens^29^, who argued that residential locations should not be treated as a neutral spatial anchor in accessibility measurement. Instead, they emphasize that where people live is shaped by constrained residential choice, which determines people’s starting points of accessibility and their mobility potential^35^. In line with this perspective, we continued to use land value as an indicator of structural context^36^, reflecting differences in neighborhood-level economic conditions that shape individuals’ ability to access and meaningfully benefit from personally valued parks.

Panel (**b**) visualized the self-identified park reported by respondents, along with their perceived cultural ecosystem service (CES) value, measured on a 5-point Likert scale. While this perceived value provided context for leisure opportunities in Jakarta, the current study did not compare green space quality across locations. Instead, we treated each location as equally meaningful to the individual who selected it and did not compare green spaces with one another. This framing ensures an equitable basis of comparison within the study: while all respondents have formal access to their chosen park, their ability to reach and use it still varies. This conceptual focus minimized the impact of inter-park variation, thereby reinforcing the analysis to focus on disparities in actual access and use.

### Multimodal travel time estimation

To estimate travel times from each respondent’s residence to their self-identified park, this study utilized various routing tools based on the transport mode. For cars, motorcycles, walking, and cycling, distance and travel time matrices were calculated using OpenRouteService (ORS) within QGIS (plugins.qgis.org/plugins/ORStools/). However, ORS does not offer a separate speed profile for motorcycles. Thus, motorcycle times were initially derived using the car profile and then adjusted by a conversion factor of 0.92, based on empirical speed comparisons between motorcycles (14.1 km/h) and cars (13 km/h) in Jakarta^37^. This factor reflects the motorcycle’s greater maneuverability and efficiency in congested urban conditions.

For public transport (PT) users, travel times were obtained using Google Maps, referencing the actual time of the reported visit. This divergence from ORS is intentional and reflects the distinct structural constraints faced by PT users—including access and egress walking, fixed routes, transfers, and waiting times—which ORS cannot simulate. Google Maps routing incorporates General Transit Feed Specification (GTFS) data from local transit agencies, including scheduled transit routes, stop locations, and timetables, enabling accurate, time-sensitive routing and realistic route availability (developers.google.com/transit).

This methodological choice enhanced the Capability Approach’s validity by avoiding mode equalization and emphasizing the lived constraints of mobility. While divergence in routing tools may challenge comparability, it reflects structural asymmetries in urban mobility: private vehicles enable direct travel, whereas public transport users face fragmented, time-consuming journeys with walking, transfers, and fixed schedules. Using Google Maps for public transport routing therefore better represented real constraints, aligning with the Capability Approach’s focus on justice in opportunities over assumed or theoretical access.

During this travel time estimation, only 377 of the original 386 records were retained as complete cases. A small number of respondents (n = 8) were excluded due to missing data on their preferred mode of transport, a critical variable for this study. One additional origin–destination pair was excluded due to limitations in the routing algorithm. These exclusions were minimal, not spatially or socioeconomically clustered, and are unlikely to introduce bias in the findings. The distribution of the final dataset, based on respondents’ self-reported preferred transport modes, is shown in Supplementary Fig. 3.

### Conversion assessment through the benefit–cost (B/C) ratio

This study introduced a time-based benefit–cost (B/C) ratio as a diagnostic of conversion efficiency, specifically, how effectively access translated into meaningful green space use. The ratio compared the realized time spent in a preferred park as a benefit with the travel time required to reach it as a cost. Respondents’ self-identified destinations and observed transport modes provided the basis for estimating travel effort, while reported visit frequency and duration captured realized use. Monthly time spent in green space was calculated as:

$$Monthly UGS Use \left(minutes\right)=Visit Frequency \left(per month\right)\times Duration (per visit)$$

For the B/C ratio calculation, travel time was treated as a round-trip measure, reflecting the full temporal burden of sustaining access. The B/C ratio was then computed for each respondent in Microsoft Excel by dividing their monthly time spent in by their respective travel time as:

$$B/C Ratio= \frac{Monthly UGS Use (minutes)}{Visit Frequency \left(per month\right)\times (2 \times One-Way Travel Time \left(minutes\right))}$$

Higher ratios indicate greater flexibility in converting access into meaningful use; lower ratios signal a constrained agency, where nominal access fails to yield substantial benefits. The resulting B/C ratios were analyzed and visualized as boxplots, stratified by socioeconomic status and transport mode, using RStudio version 2023.12.1. This intersectional assessment of constraints translated individual-level capability analysis into an operational tool for understanding how structural inequality and access to mobility options shaped conversion outcomes.

To translate individual-level conversion analysis into actionable spatial insights, B/C ratios were aggregated at the ward level and visualized using the heatmap renderer in QGIS (v3.38.3). The B/C ratio was specified as the weighting field, a rendering radius of 5 mm, automatic scaling of the maximum intensity value, and the rendering quality set to “Best”. The heatmap highlighted areas where respondents more efficiently converted green space access into actual use, as well as wards with fewer conversion barriers. This spatial aggregation operationalized the Capability Approach at a policy-relevant scale, providing a diagnostic tool to inform targeted interventions.

## Supplementary Information

Below is the link to the electronic supplementary material.

Supplementary Information.

## Data Availability

The data for this study is available at https:/doi.org/10.6084/m9.figshare.30186553. The land value data are publicly available for download^33^.

## Code availability

The source code for plotting the boxplots, usage heatmap matrix, stacked bar charts, and spatial heatmap is available from 10.6084/m9.figshare.30186658.

## References

[1] World Health Organization. *Urban Green Spaces: A Brief for Action* (World Health Organization, 2017).

[2] UN-Habitat. *World Cities Report 2020: The Value of Sustainable Urbanization* (United Nations Human Settlements Programme, 2020).

[3] Wolch, J. R., Byrne, J. & Newell, J. P. Urban green space, public health, and environmental justice: The challenge of making cities ‘just green enough’. *Landsc. Urban Plan.* **125**, 234–244 (2014).

[4] World Health Organization. *Urban green spaces and health – a review of evidence* (World Health Organization, 2016).

[5] Haase, D. et al. Greening cities – To be socially inclusive? About the alleged paradox of society and ecology in cities. *Habitat Int.* **64**, 41–48 (2017).

[6] Kabisch, N. & Haase, D. Green justice or just green? Provision of urban green spaces in Berlin, Germany. *Landsc. Urban Plan.* **122**, 129–139 (2014).

[7] Rigolon, A. A complex landscape of inequity in access to urban parks: A literature review. *Landsc. Urban Plan.* **153**, 160–169 (2016).

[8] Boone, C. G., Buckley, G. L., Grove, J. M. & Sister, C. Parks and people: An environmental justice inquiry in Baltimore, Maryland. *Ann. Assoc. Am. Geogr.* **99**, 1–21 (2009).

[9] Schlosberg, D. Ch. 2. In *Defining Environmental Justice: Theories, Movements, and Nature* (Oxford University Press, 2007).

[10] Fraser, N. Ch. 2. In *Scales of Justice: Reimagining Political Space in a Globalizing World* (Columbia University Press, 2008).

[11] Low, S., Taplin, D. & Scheld, S. Ch. 1. In *Rethinking Urban Parks: Public Space and Cultural Diversity* (University of Texas Press, 2005).

[12] Checker, M. Wiped out by the “greenwave”: Environmental gentrification and the paradoxical politics of urban sustainability. *City Soc.* **23**, 210–229 (2011).

[13] Agyeman, J., Bullard, R. D. & Evans, B. Ch. 5. In *Just Sustainabilities: Development in an Unequal World* (Earthscan Publications Ltd, 2003).

[14] Zain, A. M., Pribadi, D. O. & Indraprahasta, G. S. Revisiting the green city concept in the tropical and global south cities context: The case of Indonesia. *Front. Environ. Sci.* **10**, 1–15 (2022).

[15] Leitner, H. & Sheppard, E. From kampungs to condos? Contested accumulations through displacement in Jakarta. *Environ. Plan. A Econ. Space* **50**, 437–456 (2017).

[16] Rahman, I., Grunwald, A. & Saha, S. Access to cultural ecosystem services and how urban green spaces marginalize underprivileged groups. *NPJ Urban Sustain.* **5**, 1–11 (2025).

[17] White, M. P. et al. Spending at least 120 minutes a week in nature is associated with good health and wellbeing. *Sci. Rep.* **9**, 7730 (2019). 31197192 10.1038/s41598-019-44097-3 PMC6565732

[18] Dennis, M., Cook, P. A., James, P., Wheater, C. P., and Lindley, S. J. Relationships between health outcomes in older populations and urban green infrastructure size, quality and proximity. *BMC Public Health* **20** (2020). 10.1186/s12889-020-08762-x PMC7201616 32375720

[19] Sen, A. *Inequality Reexamined* Ch. 3 (Oxford University Press, 1992).

[20] Sheller, M. *Mobility Justice: The Politics of Movement in the Age of Extremes* Ch. 1 (Verso, 2018).

[21] Michelangeli, A., Östh, J., Toger, M. & Türk, U. Inequality in access to urban amenities. *Npj Urban Sustain.* **5**, 1–7 (2025).

[22] v. Burgsteden, M., Grigolon, A. & Geurs, K. Improving community wellbeing through transport policy: A literature review and theoretical framework, based on the capability approach. *Transp. Rev.* **44**, 1161–1186 (2024).

[23] Goodin, R. E., Rice, J. M., Parpo, A. & Eriksson, L. *Discretionary Time: A New Measure of Freedom* Ch. 5.1 (Cambridge University Press, 2008).

[24] Lucas, K. Transport and social exclusion: Where are we now?. *Transp. Policy* **20**, 105–113 (2012).

[25] Martens, K. *Transport Justice: Designing Fair Transportation Systems* Ch. 8 (Routledge, 2017).

[26] v. Wee, B. & Geurs, K. Discussing equity and social exclusion in accessibility evaluations. *Eur. J. Transp. Infrastruct. Res.* **11**, 350–367 (2011).

[27] Schwanen, T. & Wang, D. Well-being, context, and everyday activities in space and time. *Ann. Assoc. Am. Geogr.* **104**, 833–851 (2014).

[28] Nahmias-Biran, B. & Shiftan, Y. Using activity-based models and the capability approach to evaluate equity considerations in transportation projects. *Transportation* **47**, 1–19 (2019).

[29] Vecchio, G. & Martens, K. Accessibility and the capabilities approach: A review of the literature and proposal for conceptual advancements. *Transp. Rev.* **41**, 833–854 (2021).

[30] Alkire, S. *Valuing Freedoms* Ch. 1.3 (Oxford University Press, 2002).

[31] Robeyns, I. The capability approach in practice. *J. Polit. Philos.* **14**, 351–376 (2006).

[32] Satu Data Indonesia, *Data Kepadatan dan Luas Wilayah Per Kelurahan di Provinsi DKI Jakarta*, https://data.go.id/dataset/dataset/data-kepadatan-dan-luas-wilayah-per-kelurahan-di-provinsi-dki-jakarta (2025).

[33] Jakarta Provincial City Government, *Penetapan Nilai Jual Objek Pajak Bumi dan Bangunan Pedesaan dan Perkotaan Tahun 2021*, https://peraturan.bpk.go.id/Details/167231/pergub-prov-dki-jakarta-no-17-tahun-2021 (2021).

[34] Hwang, Y. H., Nasution, I. K., Amonkar, D. & Hahs, A. Urban green space distribution related to land values in fast-growing megacities, Mumbai and Jakarta–Unexploited opportunities to increase access to greenery for the poor. *Sustainability* **12**, 1–17 (2020).35136666

[35] Cheshire, P. & Sheppard, S. On the price of land and the value of amenities. *Economica* **62**, 247–267 (1995).

[36] Rigolon, A. & Németh, J. What shapes uneven access to urban amenities? Thick injustice and the legacy of racial discrimination in Denver’s parks. *J. Plan. Educ. Res.* **41**, 312–325 (2021).

[37] Widita, A., Ikaputra, & Widyastuti, D. T. The anatomy of ride-hailing trips in the Jakarta metro: spatial patterns, trip-level characteristics, and interaction with other modes. *Computational Urban Science* **4**, 1–16 (2024).

