# Supplementary material for: Advancing the capability approach to well-being pathways from access and use of urban green space
Source: Sci Rep. 2026 Jul 20;16:22713. doi: 10.1038/s41598-026-63359-5 (PMC13385391; doi:10.1038/s41598-026-63359-5)
Supplement: Supplementary file 1 — Supplementary Information. [file 41598_2026_63359_MOESM1_ESM.pdf]

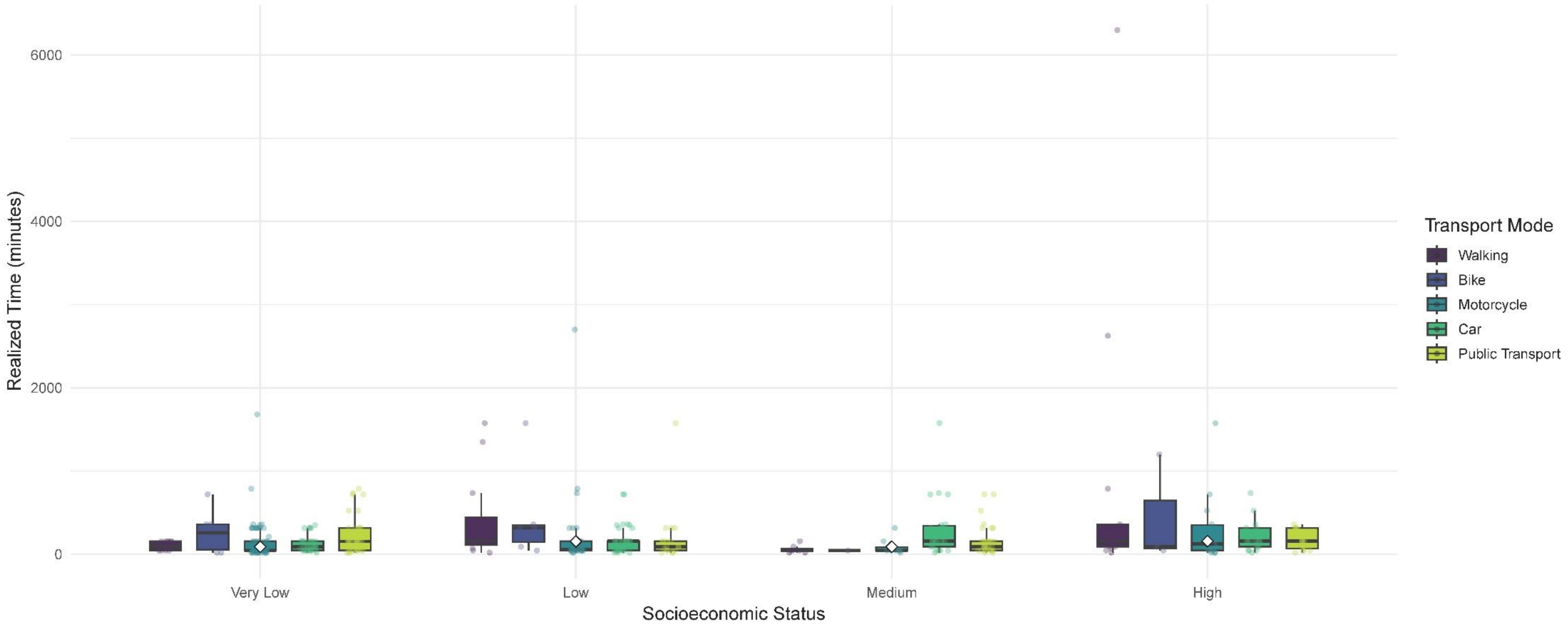

Supplementary Figure 1 | Realized time distributions by socioeconomic status (SES) and Transport Mode. The plot summarizes the reported realized time, defined as the product of monthly visit frequency and the duration of each visit. It visualizes the amount of time respondents actually spent benefiting from their preferred UGS, providing a baseline for understanding well-being outcomes. Each box represents the distribution of realized time across SES groups (x-axis) and transport modes (color-coded). Medians (white diamonds) help clarify central tendencies despite the wide spread of raw data points (faded dots).

Supplementary Table 1 | Description of survey-based and derived variables in relation to Capability Approach concepts. This table details how individual indicators, including both direct survey responses and derived measures, were aligned with Capability Approach categories of agency, conversion factors, constraints, and realized functioning.

Variable Source	Variable Used	Details or Survey Instruction / Question	Amartya Sen's Capability Approach
From the Survey: Part 1 - UGS Usage Pattern	Frequency of visit	How often do you visit urban green spaces in your city?<br>☐ once a month<br>☐ 2-5 times a month<br>☐ 6-10 times a month<br>☐ 10-25 times a month<br>☐ Everyday	Realized functioning: achieved pattern of use (frequency as outcome of access).
	Duration of visit	During your visit, how long do you stay there on average?<br>☐ Less than 30 minutes<br>☐ 30 to 60 minutes<br>☐ 60 to 120 minutes<br>☐ 120 to 180 minutes<br>☐ more than 180 minutes	Realized functioning: achieved depth of engagement (duration of use as outcome).
From the Survey: Part 2 - Cultural Ecosystem Service Mapping and Valuation from Urban Green Space	UGS Location	Pin your favorite UGS (one location)<br>[*pinpoint mapping*]	Agency/Valued choice: Park that people themselves see and value as meaningful.
	Residential postal code	Postal code of your residence<br>[*fill in the blank*]	Conversion factor (structural): the place of residence conditions how someone can act on their agency and valued choice.
	Transport Mode	What means of transportation do you use to visit this UGS?<br>◯ by walking<br>◯ by bike/scooter<br>◯ by motorcycle (including app-based ride-hailing)<br>◯ by car (including app-based ride-hailing)<br>◯ by public transportation (bus/train)	Conversion factor (mobility): Available transport options enabling or constraining the realization of agency.

Variable Source	Variable Used	Details or Survey Instruction / Question	Amartya Sen's Capability Approach
Not from the Survey	Travel time	Travel time was calculated from the centroid coordinate of the postal code area, designated as the point of origin, to the marked UGS coordinate, designated as the point of destination, using QGIS software.	Capability constraint: Travel time represents a practical barrier or enabler of realized access; excessive time costs reduce the feasibility of converting nominal access into functioning.
Not from the Survey	Socioeconomic Status (SES)	SES was obtained from the Jakarta Provincial Government's dataset for land value information at the street level (Peraturan Gubernur No. 17/2021). The data was processed alongside the indicated postal code to profile respondents' socioeconomic class.	Conversion factor (structural): SES conditions affordability, flexibility, and the capacity to act on agency. It mediates whether spatial access can practically become a usable opportunity.
From the Survey:<br>Part 3<br>Health Benefits from Green Space Visit	Reported mental state	After visiting UGS and having culturally related physical activities, I have<br>◯ felt cheerful & in good spirits<br>◯ felt calm and relaxed<br>◯ felt active and vigorous<br>◯ woke up feeling fresh and well-rested<br>◯ I do not feel any of the aforementioned mental situations	Realized functioning: achieved mental state resulting from UGS use.
	Reported mental benefit	Among health benefits obtained from green space visits, which benefit has the greatest impact on improving the quality of your physical and/or mental health?<br>◯ Improved physical fitness and functioning of the immune system<br>◯ reduced stress levels after interaction with nature<br>◯ fulfillment of social interaction needs<br>◯ improved sleep<br>◯ Others	Realized functioning: achieved health-related outcomes (physical or mental) as perceived benefits of UGS use.

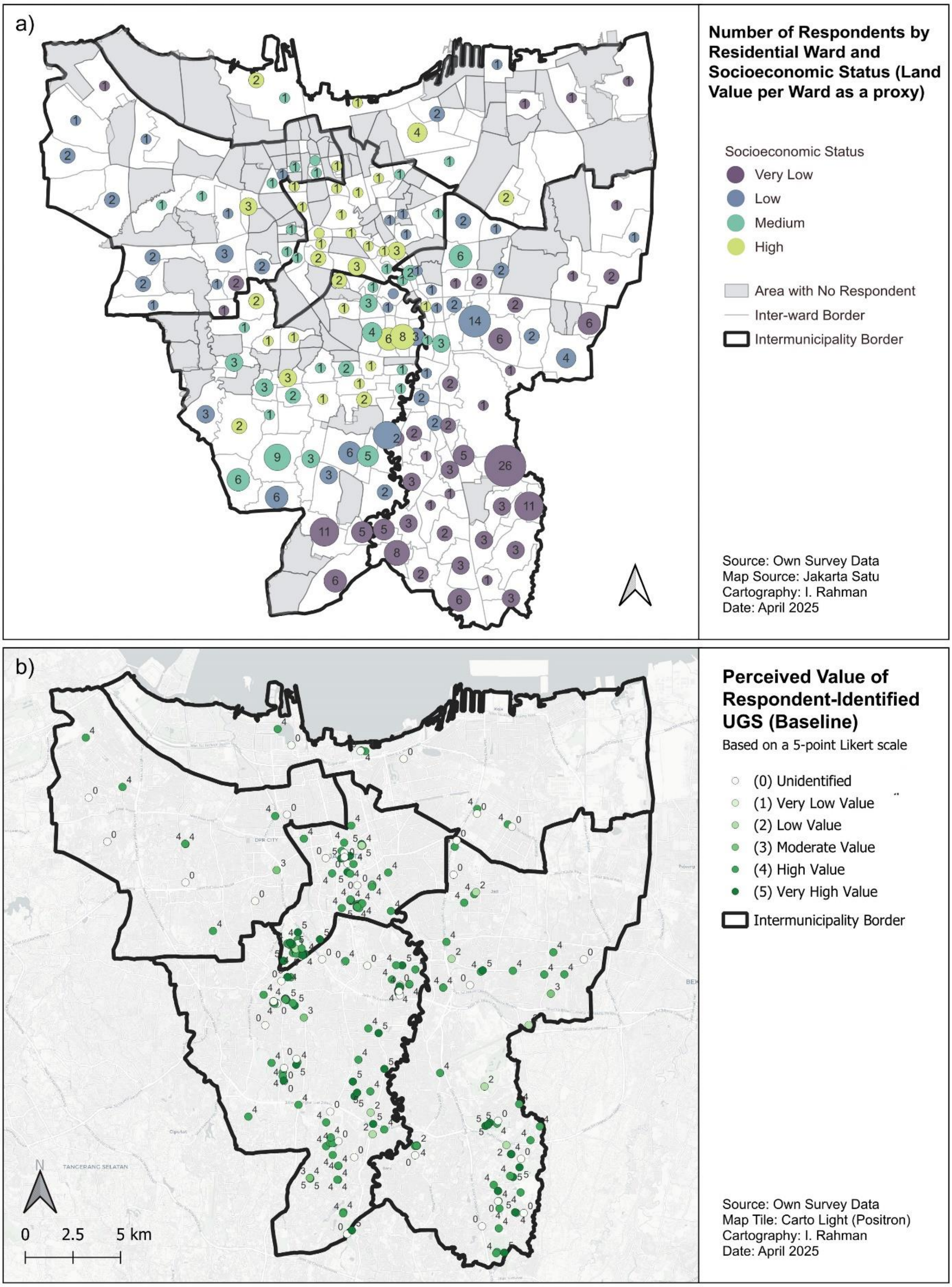

Supplementary Figure 2 | Origins of respondents and their self-reported UGS locations and perceived Cultural Ecosystem Services (CES) value. Spatial distribution of survey respondents (n = 386) across wards in Jakarta. Dot size represents the number of respondents per ward, with color indicating ward-level land value, used here as a proxy for local socioeconomic status (**a**). Variation in perceived CES value attributed to respondents' preferred UGS locations (n = 386) across the city, as reported on a 5-point Likert scale (**b**).

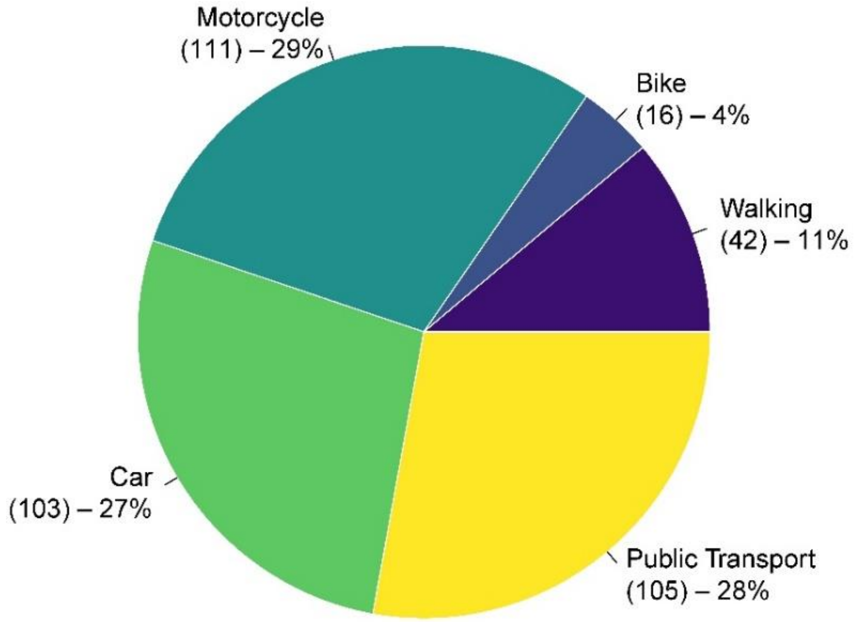

Supplementary Figure 3 | Distribution of five preferred transport modes among respondents (n = 377). Mode choice may reflect underlying spatial accessibility barriers related to respondents' socioeconomic differences in accessing their preferred UGS.
